# Stimuli-Regulated Smart Polymeric Systems for Gene Therapy

**DOI:** 10.3390/polym9040152

**Published:** 2017-04-24

**Authors:** Ansuja Pulickal Mathew, Ki-Hyun Cho, Saji Uthaman, Chong-Su Cho, In-Kyu Park

**Affiliations:** 1 Department of Biomedical Sciences, Chonnam National University Medical School, Gwangju 61469, Korea; annsaramathew@gmail.com (A.P.M.); sajiuthaman@gmail.com (S.U.); 2 Department of Plastic Surgery, Institute of Dermatology and Plastic Surgery, Cleveland Clinic, 9500 Euclid Ave, Cleveland, OH 44195, USA; tea0353@naver.com; 3 Department of Agricultural Biotechnology and Research Institute for Agriculture and Life Sciences, Seoul National University, Seoul 08826, Korea; 4 Department of Biomedical Sciences, BK21 PLUS Center for Creative Biomedical Scientists at Chonnam National University, Research Institute of Medical Sciences, Chonnam National University Medical School, Gwangju 61469, Korea

**Keywords:** stimuli-responsive system, internal stimuli, transfection, gene carrier, enzyme, temperature, polyplexes

## Abstract

The physiological condition of the human body is a composite of different environments, each with its own parameters that may differ under normal, as well as diseased conditions. These environmental conditions include factors, such as pH, temperature and enzymes that are specific to a type of cell, tissue or organ or a pathological state, such as inflammation, cancer or infection. These conditions can act as specific triggers or stimuli for the efficient release of therapeutics at their destination by overcoming many physiological and biological barriers. The efficacy of conventional treatment modalities can be enhanced, side effects decreased and patient compliance improved by using stimuli-responsive material that respond to these triggers at the target site. These stimuli or triggers can be physical, chemical or biological and can be internal or external in nature. Many smart/intelligent stimuli-responsive therapeutic gene carriers have been developed that can respond to either internal stimuli, which may be normally present, overexpressed or present in decreased levels, owing to a disease, or to stimuli that are applied externally, such as magnetic fields. This review focuses on the effects of various internal stimuli, such as temperature, pH, redox potential, enzymes, osmotic activity and other biomolecules that are present in the body, on modulating gene expression by using stimuli-regulated smart polymeric carriers.

## 1. Introduction

The rapid growth of the knowledge of gene function and gene mutations has led to a promising therapeutic strategy based on the idea of using ‘genes’ as drugs or medicine [1]. This approach was followed by the development of effective technologies for DNA delivery to mammalian cells that may help ameliorate disease conditions in three ways: introducing a new gene, replacing a mutated gene causing disease or knocking out a mutated gene [2]. Thus, the prospects of gene therapy have improved rapidly. However, because of various systemic and cellular barriers, the transfection efficiency of genes remained a challenge until the development of efficient viral and non-viral gene delivery systems; viral gene delivery systems based on vectors, such as adenoviruses, retroviruses, are successful gene delivery systems, although they have limitations, such as immunogenicity and a marked decrease in the transgenic capacity size. In contrast, the non-viral delivery systems have various advantages, such as availability, a lower induction of immunological reactions, a lack of size limitations of the genetic material and cost effectiveness, although they are comparatively less efficient in gene transduction [3,4]. Non-viral gene delivery systems include all other delivery methods, including physical methods, such as magnetofection, electroporation and gene guns, and chemical methods, such as cationic polymers and liposomes. Chemical systems, a widely-used non-viral method, generally comprise nanomeric complexes, such as lipoplexes and polyplexes formed by the compaction of negatively-charged nucleic acids with positively-charged cationic liposomes or polymers. The nanomeric complexes formed from nucleic acids and cationic liposomes are called lipoplexes, and those formed from nucleic acids and cationic polymers are called polyplexes. These nanomeric complexes protect bound nucleic acids from degradation and are easily taken up by cells [3,5]. The nucleic acids are physically complexed or encapsulated or are chemically conjugated to the non-viral gene delivery carriers [6]. Many non-viral gene delivery systems made of components such as cationic polymers, cationic lipids and lipid/polymer hybrids have been developed to overcome the safety issues of viral vectors [6]. Polymeric gene carriers are one of the best gene transfer methods because a wide range of chemical modifications of the polymeric vectors can be exploited through attachment of targeting ligands or sensitive chemical moieties, such as environmentally-sensitive linkers or bonds, to enhance transfection efficiency. For enhanced transfection efficiency, the polymeric nanovehicles must overcome extracellular barriers, such as effective blood circulation followed by extravasation across the vascular endothelial membrane, diffusion across the extracellular matrix (ECM) and association with cells and subsequent cellular uptake and intracellular processes, such as endosomal escape, polyplex unpacking and nucleic acid release in the cytoplasm or nucleus [7,8,9].

Considerable effort has been made in recent years to develop therapeutic gene carriers that can overcome these barriers and offer a better localization and controlled release at the target biological compartment in the body, as well as maintain clinical availability after their administration. This approach has been achieved by using intelligent/smart polymeric systems that adjust the therapeutic gene release in response to the physiological needs [10].

## 2. Intelligent/Smart Polymers for Stimuli-Responsive Polymeric Gene Carriers

Intelligent polymers are those that undergo changes in their conformation or properties in response to small physical or chemical stimuli in the environment [11]. They are also called ‘stimuli-responsive’ polymers because of their ability to respond to stimuli, and their uniqueness lies in their responsiveness to very slight stimuli. They undergo very fast microscopic structural transitions with reversibility, and they are biocompatible, flexible, strong and resilient and, therefore, can be easily administered in vivo [12,13]. This intelligent or smart polymeric system is also called an environmentally-sensitive polymer, and it has been studied extensively together with other materials for the on-demand release of cargo, such as drugs, gene and contrast agents [14].

A stimuli-responsive polymeric system can be devised by using a stimuli-responsive polymer or by combining a polymer with a stimuli-responsive compound, such that the polymer acts only as a carrier [15,16]. The environmental changes or stimuli, such as light, temperature, ionic strength, pH, radiation, electrical field, magnetic field and the presence of metabolic chemicals, lead to changes in physiochemical properties or structural conformations of these polymers. These specific triggers or stimuli are divided into two categories: internal stimuli, i.e., naturally-occurring variations in physiological and pathological conditions in cells or tissues (e.g., temperature, redox, enzyme activity and pH), and external stimuli, i.e., externally-induced variations in biological systems (e.g., electric field, magnetic field and ultrasound). Thus, the internal stimuli-responsive delivery system is controlled by specific inherent physiochemical parameters and the alterations that exist at the site. Unlike internal stimuli, external stimuli use many contrast agents that monitor the accumulation at the target site and are then activated from outside the body through triggers, such as light, radiation or other triggers, at the desired time [17].

**Figure 1:**
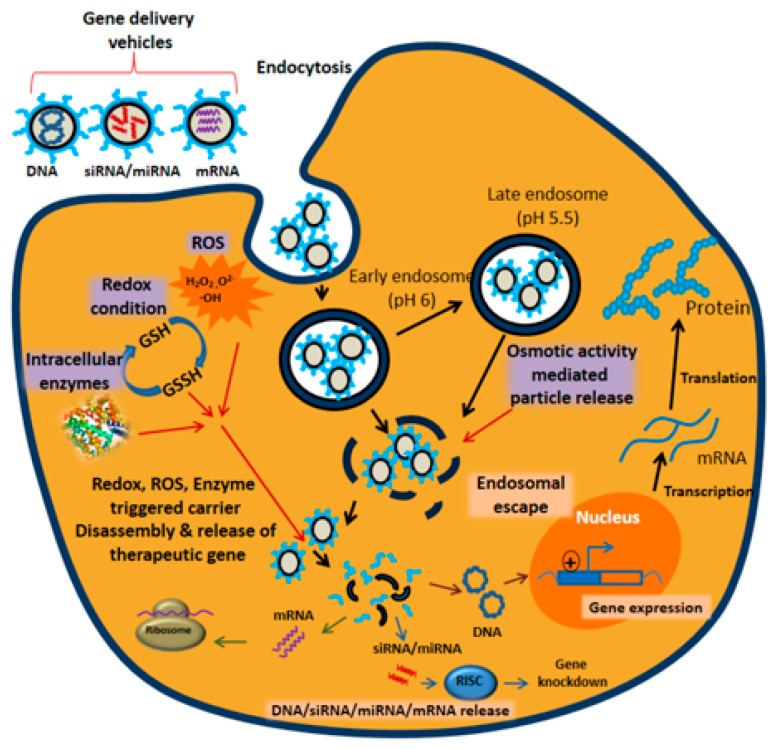
Different intracellular stimuli (ROS, intracellular enzymes, redox condition and osmotic activity) mediated release of gene carriers and subsequent gene release inside cellular environment.

Intracellular and extracellular (internal stimuli) responsive gene delivery systems have greater activity both in vitro and in vivo than their non-responsive counterparts. Figure 1 shows the role of major intracellular stimuli in mediating gene delivery. In this review, we will discuss the effects of gene expression on the polymeric gene carrier systems by internal triggers, such as pH, temperature, reactive oxygen species (ROS), enzymes, redox potential and osmotic activity. For brevity, in this review, we exclude equally important external triggers and instead will discuss only internal triggers and polymeric gene carrier system that responds to internal triggers endogenous to the target area, such as pH, temperature, reactive oxygen species (ROS), enzymes and redox potential.

## 3. Internal Stimuli-Responsive Polymeric Systems for Gene Therapy

Conventional delivery systems have advantages such as prolonged circulation and decreased adverse effects; however, most of these systems have the drawbacks of slow and deficient release profiles at the pathological sites, thus decreasing the clinical treatment benefits. Hence, it is important to develop intracellular environment-responsive delivery systems that are stable under extracellular conditions and provide good therapeutic efficacy. Maintaining the stealth property of the polymeric carrier during circulation and its transformation to a cell interactive form after reaching the target is important for effective delivery; this maintenance can be achieved by taking advantage of various extracellular environments, such as unique tumor microenvironments including the presence of tumor-specific enzymes and intracellular biological signals, such as endo/lysosomal pH [18]. For eliciting a better therapeutic effect, the drugs (siRNA, DNA, proteins or other drugs) should be delivered and released into specific sites and should reach cellular compartments, such as the cytoplasm or nucleus of the target cells.

Stimuli-responsive polymeric gene carriers that exhibit good extracellular stability and can release payloads in the target cells can be used for efficient delivery systems. The changes in the biological milieu at different biological sites in the body or during healthful or diseased states that are responsible for the release of the cargo at the target site include cells with different enzymes, overexpression of specific enzymes (e.g., chymotrypsin [19]), biomolecules maintaining redox potentials (e.g., glutathione [20]), enzymes with optimum parameters for their function (temperature and pH), the presence of reactive oxygen species (ROS) [21] and osmotic activity. Among these factors, the change in temperature is an extracellular stimulus, whereas pH and enzymes are involved in both extracellular and intracellular stimuli. The extracellular triggers involved in selective delivery to target areas include different environmental conditions in the tumor sites, such as low pH, low oxygen partial pressure and high levels of bioreductive molecules, whereas the intracellular triggers include the pH difference between cell organelles and the redox potential of the cytoplasm [22,23,24].

**Figure 2:**
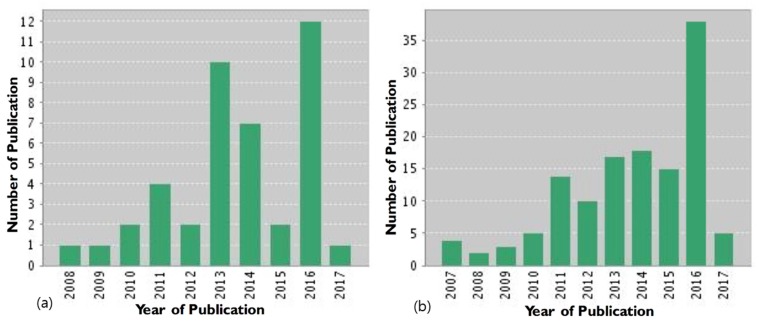
(**a**) The number of scientific papers published over the past decade on internal stimuli-based gene delivery (source: ISI Web of Knowledge: The Thompson Corporation; search terms: “internal stimuli/gene delivery”; date of search: February 2017). (**b**) The number of scientific papers published over the past decade on internal stimuli-based drug delivery systems (source: ISI Web of Knowledge: The Thompson Corporation; search terms: “internal stimuli/drug delivery”; date of search: February 2017).

Reviewing the published reports on global gene delivery for the past ten years revealed that research based on the internal stimuli-based gene delivery system is increasing. Similar trends have been reported in the field of internal stimuli-based drug delivery, which is associated with a greater number of publications, compared with gene delivery systems, as shown in Figure 2a,b.

Although many challenges exist regarding the full-scale clinical development of non-viral-based gene delivery systems, positive growth has been seen in gene delivery-based research. In this review, we discuss some of the internal stimuli-responsive delivery systems that are being developed for gene therapy.

### 3.1. pH-Responsive Systems

A pH-responsive system takes advantage of the pH variations in the body. Among the three categories of stimuli (physical, chemical and biological), pH is characterized under chemically-dependent stimuli. There are distinct pH gradients in different cells, tissues and even different cellular compartments, as well as under different physiological and pathological conditions. One example is the gastrointestinal tract (GIT) pH, which is highly acidic (pH 1–3) in the stomach, whereas there is a neutral or weak alkaline pH in the intestine (5–8). The pH also varies among normal and pathological conditions, such as wounds, in which the microorganisms change the pH to acidic or alkaline, for example a chronic wound may have a pH range of 5.4–7.4. A variety of pH gradients exist in the intracellular compartments, such as the Golgi apparatus at pH 6.4, endosomes at pH 5.5–6, lysosomes at pH 4.5–5 and the cytosol at pH 7.4 [25,26,27]. The pH difference between normal and tumor tissue is another context in which the tumor tissue possesses an extracellular acidic pH (6.0–7.0) relative to the normal physiological pH (7.4). This differential occurs because of the ineffective vascular system leading to inadequate nutrients and oxygen supply to these regions and results in the fast-growing cancerous cells undergoing glycolysis and subsequent production of lactic acid, thus making the tumor environment more acidic than the blood pH. This process of bypassing oxidative phosphorylation under anaerobic conditions is often called the Warburg effect [28,29,30]. Additionally, pH conditions within a tumor area also vary. For example, the necrotic area in the tumor becomes lower than that in actively-growing tumor regions [31]. These pH variations can be exploited in the development of a pH-responsive localized delivery system using pH-sensitive polymers. pH-sensitive, carrier-mediated release of the cargo can be controlled either by carrier destabilization or by the degradation of the pH-sensitive linker that conjugates the cargo to the carrier. Conjugation of the acid labile-linkers such as acetal [32], ketal [33], hydrazone [34], vinylester [35] and orthoester [36] onto the carriers affects pH-sensitive structural conformation or their hydrophobicity or hydrophilicity after a pH change [37].

The pH responsiveness of a polymer is based on many factors and varies according to the polymeric system. Most of the polymers used are polyelectrolytes that induce very rapid phase transitions within very small pH variations [38]. They are weakly basic (e.g., ammonium salts) or acidic (e.g., carboxylic or sulfonic) or have ionizable moieties attached to a polymeric backbone. Ionization of these pendant basic or acidic groups leads to extension/expansion of the polymer chains, owing to electrostatic repulsion [39,40]. The pH response also arises from protonation/deprotonation events, on the basis of charge distribution over these ionizable groups [41]. These polymers have natural, as well as synthetic origins, and some examples of pH-sensitive polymers include albumin, gelatin and chitosan. Chitosan is a cationic polysaccharide with a pKa of 6.2 and is used in many gene delivery applications [42]. Some other examples include poly(acrylic acid) (PAA), poly(l-lysine) (PLL), polyethylenimine (PEI), poly(methacrylic acid-*g*-ethylene glycol) (P(MAA-*g*-EG)) and poly(*N*,*N*-diakylaminoethylmethacrylates) (PDAAEMA) [11].

The pH-responsive polymers with acidic groups include PAA, with a dissociation constant of pKa 4.25, above which its carboxyl group is ionized and swells, whereas cationic polyelectrolyte poly (*N,N*-dimethylaminoethyl methacrylate) (PDMAEMA) shows ionization behavior at low pH and can be readily reversed by the pH change. Polymers containing pendant acidic groups, such as polyacid polymers, show the opposite effect. Poly(carboxylic acids), such as PAA, poly(methacrylic acid) (PMA) and polysulfonamides, are some examples of pH-sensitive polymers with anionic groups [43]. Cationic polyelectrolytes, such as chitosan, PLL, poly(*N,N*-dialkyl amino ethyl methacrylate) and PEI, show swellable properties at acidic conditions, owing to internal charge repulsion, whereas the size of the polymer decreases at higher pH values due to the polymer interaction. Different polymeric structures developed for therapeutic delivery include micelles, nanoparticles, vesicles, hydrogels, polymer brushes and dendritic polymers. The pH-sensitive polymeric systems developed for gene delivery mainly include liposomes and cationic polymers, which condense the plasmids and facilitate transport in the cells to avoid extracellular degradation [44,45].

**Figure 3:**
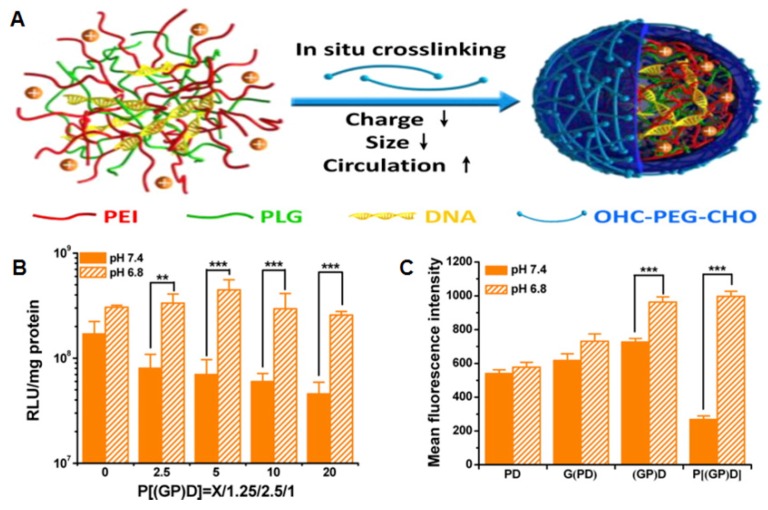

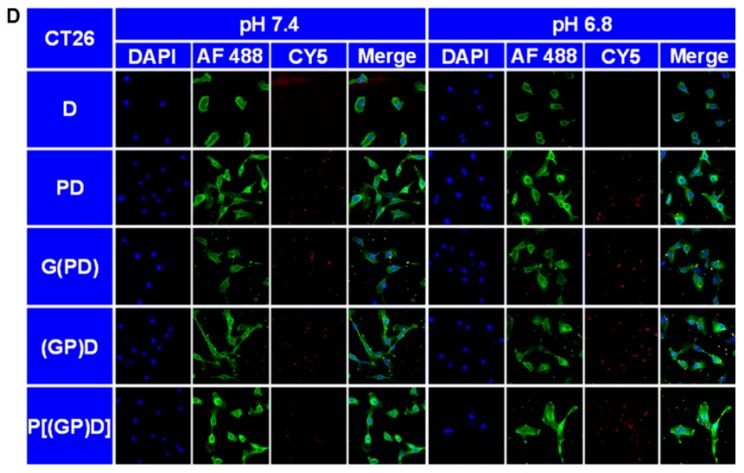
(**A**) Schematic of the pH-sensitive charge/size dual-rebound gene delivery system. (**B**) Transfection efficiency of PEG((PLG/PEI)/DNA) [P((GP)D)] with various PEG mass ratios at different pH values (7.4 and 6.8) in CT26 cells for 2 h. (**C**) Mean fluorescence intensity of cellular uptake of polyethylenimine (PEI)/DNA (PD), poly(l-glutamate) (PLG)/(PEI/DNA) (G(PD)), (PLG/PEI)/DNA((GP)D) and PEG((PLG/PEI)/DNA)(P(GP)D) at different pH values (7.4 and 6.8). (**D**) CLSM images of CT26 cells incubated with D, PD, G(PD), (GP)D and P((GP)D) at different pH values (7.4 and 6.8); Cy5-DNA was tracked. Reproduced with permission from [46]. Copyright proceedings from the American Chemical Society, 2015.

One of most important candidates used for gene delivery is PEI, and it is used to develop an ultrasensitive pH-triggered gene delivery system based on PEI and poly(l-glutamate) (PLG) generating Schiff-base bonds that are cleavable at a specific tumor pH. PEI was developed for tunable gene delivery. Through this process, a plasmid DNA that expresses small hairpin RNA targeting VEGF was used as the therapeutic gene. The negatively-charged pDNA was complexed with PEI and PLG to form the gene-loaded polymer complex NP, which was later tightened with PEG with aldehyde groups at both termini. The Schiff base formed between the aldehyde group of PEG and the amino group of PEI was stable at physiological pH (7.4), but cleavable in acidic pH, thus leading to shedding of the PEG shielding. Figure 3 shows a schematic illustration of the pH-sensitive gene delivery system and its transfection efficiency in mouse colon carcinoma CT-26 cell line according to different mass ratios [46].

Kataoka et al. have developed a polyplex structure and a charge conversion moiety and used them to form ternary polyplexes with elevated transfection efficiency and low toxicity. The positively-charged polyplex surface was covered with a degradable amide-derivatized polymer (pAsp (DET)) to form ternary polyplexes, including plasmid DNA/polycation/polyanion with the degradable side chain (DNA/pAsp (DET)/pAsp (DET-Aco). In the extracellular region, these ternary polyplexes maintain a neutral to negative charge, whereas the charge-conversion components become positively charged under the acidic milieu of the endosome, disrupt the endosomal membrane and facilitate the endosomal escape of the polyplexes. The efficiency of these ternary polyplexes has been examined in sensitive primary cells (HUVECs) and has been found to achieve a higher transfection efficiency together with low toxicity [47,48]. Use of the membrane destabilizing strategy by anionic polyelectrolyte-mediated cytoplasmic delivery of biomolecules is another strategy. These polymers mediate stimulation and acidification of the surrounding medium, thus leading to a more active interaction with the phospholipid membrane and more DNA uptake in cells [49]. The buffering capacity (pH 5.0–7.2) of polymers such as PEI and PLL leads to endosomal rupture by increasing the osmotic pressure and release of the internal content to the cytoplasm. This property of the polymer can be enhanced by chemical modifications with moieties such as cholesterol and heparin [50]. Another strategy for effective transfection is complexing pH-responsive polymers and liposomes [51]. Hydrogels are another important gene delivery system formulated with polymers such as gelatin and alginate, and they act as a gene reservoir for the localized delivery of genes [52,53]. Thus, by using the local pH changes at different sites, the stimuli-responsive system can control its distribution in the host site. Table 1 summarizes some examples of pH-sensitive gene delivery systems.

### 3.2. Redox-Responsive System

The redox-responsive gene delivery system is one of the most important stimuli-responsive systems, owing to the differences in the redox environment between intracellular and extracellular conditions. There are several redox processes that occur in the normal physiology of the intracellular environment, such as the oxygen/superoxide (O_2_/O_2_^–^) system, the nicotinamide adenine dinucleotide phosphate (NADP^+^/NADPH) system, the glutathione (GSH/GSSG) system and the thioredoxin TrxSS/Trx (SH) 2 system. Among these systems, the glutathione disulfide/reduced glutathione (GSSG/GSH) system is the most important, because of its high concentration in intracellular compartments. The intracellular GSH in tumor cells is much higher (100–1000-fold) than the extracellular levels [63]. This difference in the redox environment between the intra-cellular and extra-cellular site favors the development of redox-responsive polymeric systems. The GSH concentration in tumor sites is higher than that in normal tissues. The intracellular GSH concentration facilitates the degradation of many chemical linkages, so gene carriers have been developed to be stable in the extracellular conditions and to release the cargo only after their internalization [64]. The most important and most widely-used redox-sensitive linker for a redox-sensitive system is the disulfide linker, which is stable under low reduction conditions and is reduced into thiol groups under high reduction conditions [65]. The redox-sensitive gene delivery system should have high plasma stability to prevent leakage of the therapeutics before the target is reached and should have a relatively simple structure. Incorporation of covalent disulfide linkages on the polymeric chain can make the carrier more stable and thus prevent primitive release of DNA. The therapeutic gene is released into the cells as a result of the disulfide bond reduction, owing to the very low reduction potential in cells [66]. Thiols can be either directly introduced by reacting with disulfide containing reagents or indirectly introduced during synthesis through a thiol oxidation reduction exchange reaction. Some examples utilizing redox mechanisms for developing a gene delivery system are described in this review.

**Figure 4:**
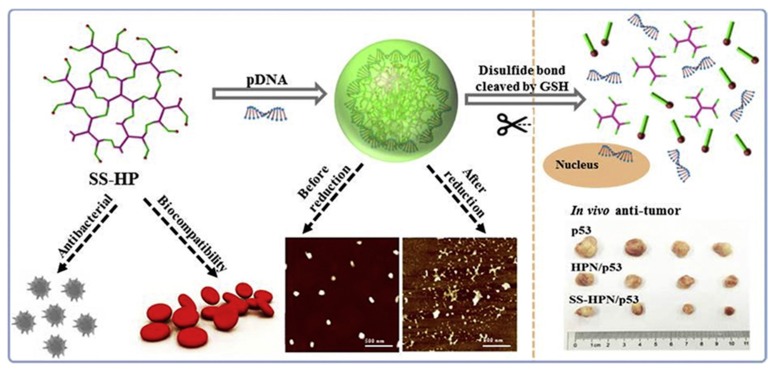
Schematic illustration of multifunctional aminoglycosides-based hyperbranched polymers (HPs) with antibacterial activity, biocompatibility and gene transfection capability. Reproduced with permission from [80]. Copyright proceedings from Biomaterials, Elsevier, November 2016.

A polycationic gene carrier using polyaspartamide has been developed by Cavallaro et al. by introducing thiol groups into the polymeric chain for disulfide bridge formation, and the positively-charged groups in the polymer interact with the negatively-charged groups in the DNA and lead to stable thiopolyplexes [67]. A thiolated gelatin nanoparticle-mediated system is another successful nanocarrier that responds to a high glutathione level. This system was developed by modification of the amino groups of gelatin by using 2-iminothiolane to obtain high transfection in NIH-3T3 cell lines through fast release of DNA from the polyplexes under a reducing environment. A modification of thiolated gelatin nanoparticles with PEG has been developed by another group and used for transfection studies. Later, this modified gelatin carrier was used for the targeted delivery of vascular endothelial growth factor receptor1 (sVEGFR-1 or sFlt-1), which shows good tumor suppression, as well as decreased angiogenesis [68,69]. Other polymers, such as thiol-modified PEI, are also widely used for gene delivery. A glutathione-sensitive cross-linked PEI (CLPEI_50%_) has been developed by cross-linking low molecular weight PEI and dimethyl 3,3′-dithiopropionimidate dihydrochloride (DTBP). CLPEI_50%_ induced DNA condensation into spherical nanoparticles and shows sensitivity to reductive glutathione (GSH). The formed CLPEI_50%_-DNA polyplexes are unpacked at a GSH concentration of 3 mM, which is similar to the intracellular environment, and show very low toxicity [70]. Another modified novel gene delivery system based on a bioreducible branched poly-CPP (cell-penetrating peptides) structure responds to reducing conditions and has been developed as both a pDNA and siRNA delivery platform. A branched-mR9 (B-mR9) has been developed by modification of nona-arginine (mR9) by disulfide bonds, and both the branched-mR9/pDNA and B-mR9/VEGF siRNA polyplex system exhibit redox-cleavability, outstanding delivery effectiveness and selective gene release in carcinoma cells. Moreover, the B-mR9 polyplexes also show outstanding tumor accumulation and inhibition ability in vivo [71]. By immobilizing thiol groups on the chitosan backbone, thiol-modified chitosan (chitosan-thiobutylamidine) has been developed and used for gene delivery applications. The thiol group immobilization leads to the formation of reversible disulfide bonds, thus improving the stability of the carrier in extracellular conditions, as well as the release of pDNA from the carrier under intracellular conditions. In the intracellular environment (reducing environment), chitosan-thiobutylamidine-DNA nanoparticles have been found to undergo continuous dissociation, to liberate approximately half of the pDNA in less than 4 h and to result in enhanced transfection efficiency in Caco2 cells [72]. Later, HA-coated stents were immobilized with microRNA145/ssPEI nanoparticles that alleviated in-stent restenosis in a rabbit restenosis model. The ssPEI developed by disulfide cross-linking of low molecular weight PEI is reduced and degraded in the intracellular environment and mediates the release of miR-145 [73]. Disulfide-linked PEI-coated gold nanoparticles (ssPEI-GNPs) prepared and used as a non-viral gene delivery system have been found to be effective in NIH-3T3 mouse embryonic fibroblast transfection [74]. Bioreducible ssPEI has also been used for the delivery of siRNA modified with thiol (Akt1-SH-siRNA) by utilizing the ability to form a disulfide bond between thiol-modified siRNA (SH-siRNA) and ssPEI. The Akt1 SH-siRNA has been found to suppress the Akt1 mRNA, decrease cellular proliferation and induce apoptosis in an in vivo mouse tumor model [75]. The disulfide bonds are also important for the stability of liposome-based gene carriers. Similarly to other polymeric systems, the reduction of disulfide bridges in liposomes also occurs in the presence of glutathione and leads to the destabilization of the liposomes and intracellular delivery of therapeutic genes. Thiocholesterol-based cationic lipids (TCL) used in liposomes for DNA encapsulation produce a lipoplex formation that releases the genes even under low amounts of reducing agents [76]. A disulfide-linked PEG coating on liposomes has been found to further increase the circulation and enhance accumulation, as well as the release of the genes after a redox stimulus [77]. Particle replication in non-wetting templates (PRINT) is a high-resolution molding technique for the development of micro- and nano-particles with precise control over parameters such as size, shape and composition. Through a PRINT-based continuous particle fabrication strategy, highly efficient, reductively-responsive hydrogel nanoparticles with a very good loading efficiency and loading ratio for in vivo delivery of siRNA have been developed by Ma and co-workers. The thiol-modified coagulation factor VII siRNA (FVII-siRNA) was conjugated via a disulfide linker onto the nanoparticles, and this approach enables the reduction-sensitive release of the payload. The siRNA-conjugated nanoparticles showed successful gene silencing in liver hepatocytes, and both mRNA and protein levels have been shown to be decreased in these cell lines [78]. Another group has developed PRINT-mediated hydrogel nanoparticles for siRNA delivery and has shown effective knockdown of gene expression in HeLa cells. The siRNA has been shown to be effectively protected and to be released under the reducing conditions of the cytoplasm [79]. Huang et al. have prepared multifunctional reduction-responsive aminoglycosides-based on hyperbranched polymers (HPs) with excellent antibacterial activity, biocompatibility and gene transfection efficiency. They have prepared both reduction-responsive and non-responsive systems (HP and SS-HP) and used them for the delivery of p53 DNA in both in vitro and in vivo systems [80]. The schematic illustration explaining the reduction-responsive hyperbranched polymer system is shown in Figure 4.

Supramolecular systems with different moieties held together by reversible, directional and non-covalent binding interactions, an important class of biomaterial developed and applied for biomedical applications. An example is the supramolecular inclusion complex formed by cyclodextrins (CDs) with various polymers. Supramolecular self-assembly systems based on the electrostatic interaction between CDs and guest polymers such as low molecular weight PEI have been designed and used successfully as nonviral gene vectors with good cytocompatibility and enhanced transfection activity. Feng et al. developed a cyclodextrin-based cationic star polymer carrier by conjugating oligoethylenimine (OEI) with γ-CD (γ-cyclodextrin) to form γ-CD-OEI and further modification with the folic acid (FA) moiety by using bioreducible disulfide bonds (γ-CD-OEI-SS-FA1.3) for the efficient delivery of pDNA to folate receptor positive cells. The γ-CD-OEI-SS-FA1.3 gene carrier showed low toxicity and enhanced targeting to folate receptor-positive tumor cells. This study also facilitated an enhanced and continuous FR-mediated endocytosis and achieved very high levels of gene expression [81]. Another strategy for the development of multifunctional delivery system was developed by using adamantyl-functionalized polymers (PEI-Ad and Ad-PEG) and poly(β-cyclodextrin) (poly(β-CD)), where the polyplexes are formed by electrostatic interactions between PEI-Ads and the nucleic acids, and later, the PCD stabilizes the complexes. Later, Jiang et al. utilized supramolecular assembly approach for beta-cyclodextrin-based polymer (PBCD) and mono-adamantane terminated functionalized polymers by utilizing simple host-guest interactions, which then condense the DNA or siRNA to form polyplexes. The formed supramolecular polyplexes were then PEGylated by using Ad-PEG (or Ad-SS-PEG) and used for targeted gene delivery [82,83]. Various redox-sensitive gene delivery systems have been established and are listed in Table 2.

### 3.3. ROS-Responsive Systems

Reactive oxygen species (ROS) are oxygen-derived chemical species produced by the body as a result of many physiological processes. They include hydrogen peroxide (H_2_O_2_), singlet oxygen (^1^O_2_), superoxide (O_2_•^–^) and hydroxyl radicals (HO•), and ROS can be transformed through a cascade reaction [93]. ROS are maintained at a low concentration for normal cell functions and are maintained by the various antioxidants present. A higher ROS concentration leads to oxidative stress that suppresses the antioxidant defense system and induces many pathogenic conditions, such as irreversible damage to molecules such as DNA, protein and lipids, thus leading to cancer or other diseases. This change in ROS can be used for the development of ROS-responsive carriers. The ROS response leads to changes in phase transitions, hydrolysis, degradation or the solubility of polymers [94]. Different types of ROS-responsive materials, including thioketal, polysaccharide and thioether, have been explored for the delivery of therapeutics.

Liu et al. have developed a charge reversal (positive to negative) theory-based gene carrier that responds to ROS and releases the packaged DNA. In response to ROS, the polymer poly((2-acryloyl) ethyl (*p*-boronic acid benzyl) diethylammonium bromide) (B-PDEAEA) undergoes oxidation of the boronic acid group, and the quaternary ammonium became a tertiary amine, catalyzed the ester group hydrolysis and produces poly(acrylic acid). The highly positive B-PDEAEA/DNA polyplexes have further been coated with a lipid envelope that enhances the cell membrane fusion and favors the ejection of polyplexes into cytosol-like virus. These fusogenic lipoplexes (FLPPs) effectively avoid lysosomal trapping and have also been modified by PEG and tumor homing peptide (CRGDK), thereby enhancing stable circulation, tumor accumulation and therapeutic efficacy by using the TRAIL gene. After intravenous injection, the FLPPs accumulate in tumors, and the RGDK ligands of the particle bind the cell membrane receptors and eject the polyplexes into the cytosol. The intracellular ROS oxidize the B-PDEAEA, leading to a charge reversal and releasing DNA, which enters the nucleus for transcription. This gene carrier has been found to successfully condense plasmid DNA at very low N/P ratios and shows a markedly improved charge reversal rate. The in vivo therapeutic efficacy study in the A549 tumor xenograft model has reported a 70% tumor inhibition rate [95]. Another important linker is the thioketal linker, which is readily cleaved by ROS and is utilized in delivering therapeutics to sites, such as those of inflammation or cancer. A cationic poly(amino thioketal) (PATK) has been conjugated with DNA for gene delivery to prostate cancer cells [21]. The thioketal linkage is cleaved in an ROS environment and leads to DNA release and high transfection. The addition of GRP78 protein-targeting peptide on PATK enhances the cell uptake and transfection efficiency.

Thioketal-containing polymers called thioketal nanoparticles (TKNs) have been developed from an ROS-responsive polymer, poly-(1,4-phenyleneacetone dimethylene thioketal) (PPADT). The ROS-responsive thioketal linkage is stable in acidic, basic and enzyme-catalyzed reactions, but is selectively degraded in the presence of ROS. The TKNs form complexes with TNF-α siRNA, and the siRNA is released in the presence of unusually elevated levels of ROS in the intestinal inflammation sites. The orally-delivered siRNA-TKN complexes localize to inflammation sites in the intestine and inhibit the gene expression in an ulcerative colitis model, shown on the basis of decreased levels of mRNA detected in the colon. The target effect at the intestinal inflammation site, as studied in an ulcerative colitis model, is a decrease in TNF-α messenger RNA levels in the colon [96]. Yu et al. have developed an ROS (H_2_O_2_)-responsive block copolymer (mPEG-PS) composed of methoxy poly(ethylene glycol) (mPEG) and poly(diethyl sulfide) (PS), which facilitates endosomal escape of the therapeutics in cancer cells. This hydrophobic/hydrophilic exchange of the polymer has been used for efficient delivery of drugs/genes to cancer cells [97]. Most of the ROS-mediated gene delivery systems use a photosensitizer and an external laser that activates the photosensitizer for enhanced ROS generation. The photosensitizer-mediated ROS generation is not discussed in this section, because we considered only naturally-occurring or inherent ROS signals for gene delivery.

### 3.4. Enzyme-Responsive Systems

Enzymes are an important category of biological stimuli and are important constituents in the metabolic process. They are very selective and specific, and they act only with selected substrates and are also active under very mild conditions. High levels or activity profiles of specific enzymes can be used for site-specific triggered release of therapeutics by formulating an enzyme-specific carrier system. In response to the enzymes, the polymeric shells are degraded, thus resulting in the release of the therapeutics at the site. Enzyme-responsive polymeric systems can be designed by either using enzyme-degradable polymers or by modifying polymers with moieties that are responsive to specific enzymes. Major polymeric materials utilized for enzyme-responsive systems include natural polymers, such as chitosan, pectin, dextran and cyclodextrin, as well as synthetic polymers, such as poly(*N*-isopropylacrylamide) (PNIPAAm), PLL and PEG. Some of the enzymes include reductive enzymes and hydrolytic enzymes, such as human leukocyte elastase (HLE), cancer-associated proteases (CAPs), metalloproteinase, trypsin, thermolysin and elastase [98,99,100,101,102].

**Figure 5:**
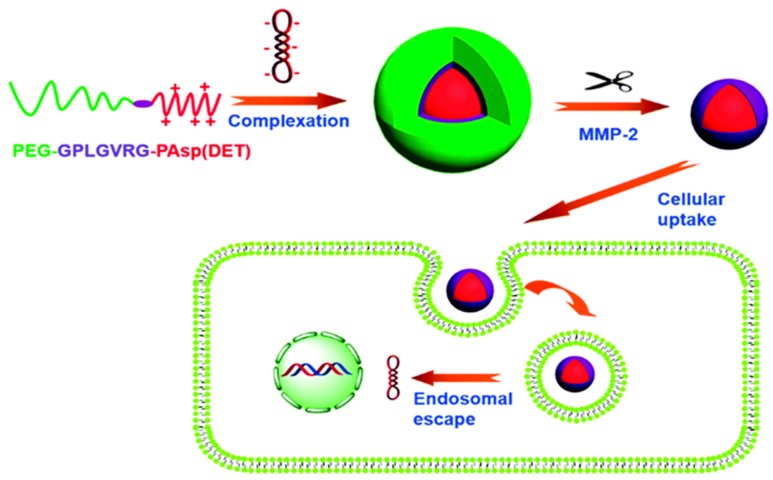
Schematic illustration showing PEG-coated polyplex micelles in MMP-2-expressing tumor tissue showing enhanced cellular uptake and endosomal escape for gene transfection. Reproduced with permission from [107]. Copyright proceedings from The Royal Society of Chemistry.

Enzymes, such as α-chymotrypsin (hydrolase), have been utilized for the preparation of enzyme-sensitive drug carriers. A layer-by-layer (LbL) self-assembly of DNA and PLL on porous CaCO_3_ microparticles has been generated for the synchronized delivery of DNA and dextran. α-chymotrypsin is a pancreatic enzyme found in many mammals, and its high concentration in the pancreas facilitates effective delivery of payloads at the target destination. The effects of the α-chymotrypsin concentration on the deconstruction of the microcapsule studies have indicated that the degradation rate of the microcapsule is directly proportional to the enzyme concentration. This dual carrier has been used for the simultaneous delivery of DNA and dextran as a model system and with proper selection of both therapeutics; this carrier system can be used for improving the therapeutic efficacy of diseases [103]. Another important class of enzymes widely used for a responsive system is the matrix metalloproteinases (MMPs). MMPs are a class of endopeptidases that specifically cleave peptide bonds between non-terminal amino acids and are overexpressed in inflammation and cancer. Incorporation of peptide substrates suitable for MMPs into the polymer can act as enzyme cleavable sites and aid in modifying the structure and morphology of the polymer system [104,105,106]. An MMP-responsive PEG-sheddable polymeric micelle gene delivery system has been prepared by diblock copolymer PEG227-GPLGVRG-PAsp (DET)_64_, as shown in Figure 5. This copolymer consists of two hydrophilic parts: one part is diethylenetriamine-modified poly(aspartamide) PAsp(DET), which is positively charged and binds the plasmid DNA to form polyplexes, and the other part is PEG, which links a GPLGVRG peptide specific for the MMP. This system forms a core-shell structure in which the PAsp (DET)/DNA compose the core structure, which is surrounded by PEG shells. Exposure of the polyplexes to MMPs results in cleavage of the peptide linkages and shedding of the PEG, thus exposing the core, which is highly positively charged and is easily taken up in the cells, thereby enhancing the transfection efficiency [107].

Zhu et al. have developed an enzyme-responsive carrier for the simultaneous delivery of drugs and genes for cancer therapy. They have used hollow mesoporous silica (HMS) and enzyme-sensitive PLL polymer and have prepared HMS/PLL particles in a layer-by-layer assembly method by using an electrostatic interaction between genes and PLL polymers. The HMS/PLL particles in the presence of α-chymotrypsin solution show simultaneous release of both fluorescein and CpG ODN, because of the degradation of PLL polymer [19]. The particles can be thus be used for the simultaneous delivery of gene and drugs and have promising applications in biomedicine and therapy.

Stabilized plasmid-lipid particles (SPLPs) are an advanced version of a liposomal system with good encapsulation and stabilization. However, the high stability of these systems results in low transfection efficiency. Therefore, enzyme-responsive SPLPs (eSPLPs) using a peptide linker GFLG (Gly-Phe-Leu-Gly) have been developed to increase the transfection efficiency. The peptide linker is placed between PEG and K (Lys) to form PEG-GFLG-K-(lipid) 2, and the palmitic acid anchor is linked to the liposome. The GFLG linker is sensitive to an endo-lysosomal enzyme cathepsin B, thus leading to shedding of the PEG shell and endosomal escape of SPLPs. This system allows for increased transfection, thus suggesting that it may be a prospective gene delivery vehicle for cancer therapy, because cathepsin B is associated with tumor progression [108,109]. By utilizing the activity of cyclic AMP-dependent protein kinase (PKA) or caspase-3, two cationic graft-type copolymers (PAK and PAC) have been developed as a signal-responsive gene-regulation system in the intracellular environment. The PAK polymer contains a receptor for PKA signals in the form of a substrate oligopeptide (ARRASLG), and the PAC polymer consists of an oligopeptide substrate sequence forcaspase-3 (DEVD) and a cationic oligolysine, KKKKKK. The PAC-DNA or PAK-DNA complexes are disassembled in the presence of caspase-3 or (PKA), thereby leading to activated gene expression [110]. Thus, this strategy can be used to develop a gene delivery system based on a wide range of intracellular signals, because this type of complex can be actively disassembled in response to many target cellular signals. Apart from the development of all these enzyme-responsive systems, the enzymes utilized for these systems are limited, and the uncontrolled rate of an enzymatic system also hinders effective delivery.

### 3.5. Osmotic-Responsive System

Several factors, such as physical, geometrical, chemical and biological cues, affect the regulation of cellular uptake. The osmotic-responsive system is one physical cue. The physical cues are physical stimuli obtained by non-viral gene carriers that trigger the regulation of cellular uptake.

Cho and co-workers have demonstrated that osmotically-active polymeric gene carriers increase cellular uptake and result in increased gene expression [111], although Maiti et al. first reported that a sorbitol-based molecular transporter can function as one of the osmolytes that enhances cellular uptake [112], and similarly, Higashi et al. have reported increased transfection efficiency of the plasmid DNA and enhanced gene silencing of siRNA [113] without providing an explanation of the exact mechanism of these transporters. Interestingly, it has already been reported that cells exposed to the osmotically-active conditions selectively stimulate caveolae-mediated endocytosis (CME) by downregulation of the clathrin-mediated endocytosis (CDE) and micropinocytosis [114]. The CME pathway also follows a non-digestive and non-acidic route without lysosomal fusion and degradation.

**Figure 6:**
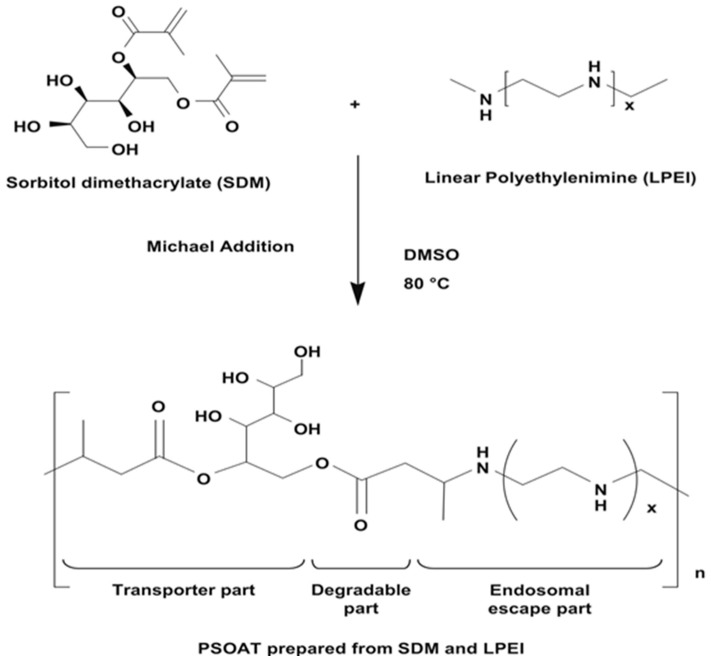
Schematic representation of the synthesis of PSOAT. Reproduced with permission from [115]. Copyright proceedings from Elsevier.

**Figure 7:**
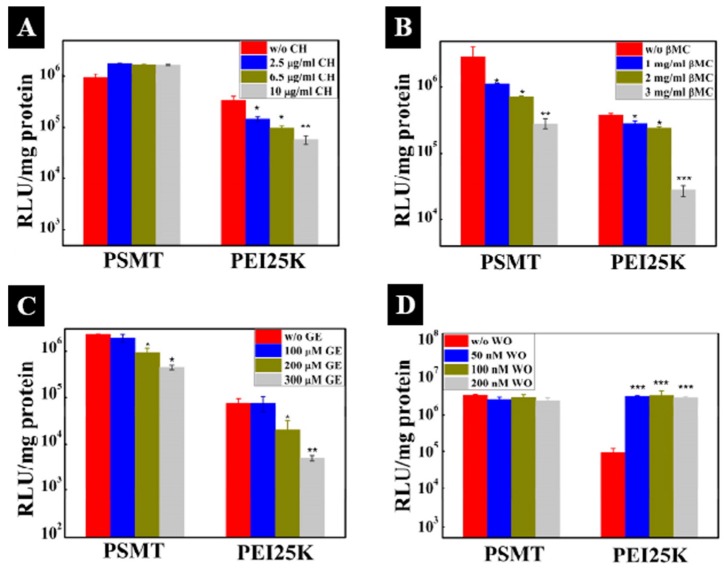
Effects of (**A**) chlorpromazine, (**B**) β-methyl cyclodextrin, (**C**) genistein and (**D**) wortmannin on transfection efficiency in A549 cells. Reproduced with permission from [116]. Copyright proceedings from the American Chemical Society.

Cho’s group has confirmed that the hyperosmotic poly(sorbitol-*co*-PEI) gene transporter prepared by low molecular weight PEI (MW: 423, 600, 1200 and 1800) cross-links with sorbitol diacrylate (or dimethacrylate) through a Michael-addition reaction, as shown in Figure 6. This system has a high transfection efficiency of DNA [115,116,117] and results in high gene silencing by siRNA [118,119] or microRNA [120,121], owing to the selectively-directed cellular uptake pathway of the polyplexes to the CME, as shown in Figure 7.

**Figure 8:**
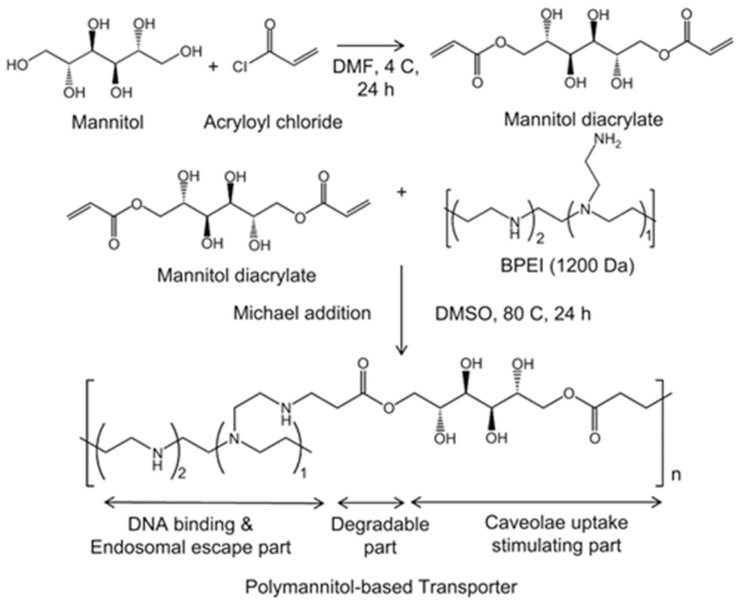
Schematic representation of the synthesis of poly (mannitol-*co*-PEI) (PMT). Reproduced with permission from [122] Copyright proceedings from Elsevier).

**Figure 9:**
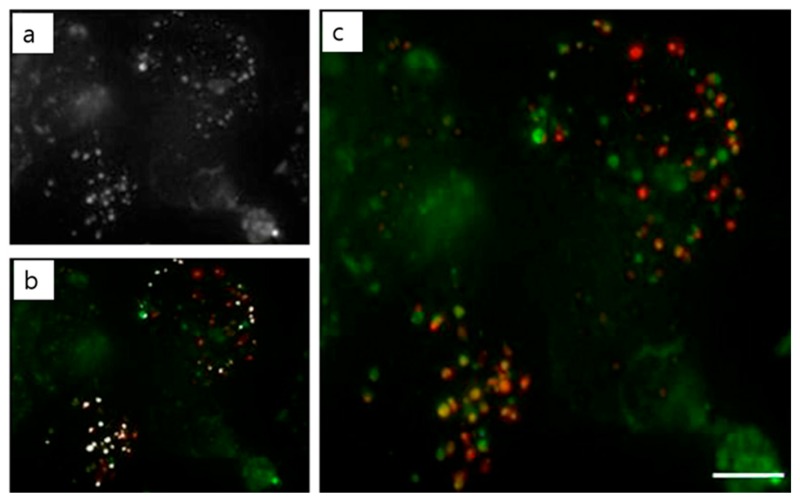
Fluorescent microscopy images showing lysosome staining (red) and FITC-PEI/DNA polyplexes (green); (**a**) Polyplex with FITC (**b**) Colocalization spot using the Image J program; (**c**) overlay of lysosomes and polyplexes (yellow). Scale bar represents 5 μm. Reproduced with permission from [122]. Copyright proceedings from Elsevier).

Cho’s group has also reported that another hyperosmotic poly(mannitol-*co*-PEI) (PMT) gene transporter obtained by low molecular weight PEI (MW: 1200) cross-links with mannitol diacrylate, as shown in Figure 8 [122], thus indicated high osmolarity, as shown in Table 3, and high transfection efficiency of DNA, owing to the avoidance of polyplexes from lysosomal fusion, as well as degradation by selective regulation of the cellular uptake route toward CME, as shown in Figure 9 [122]. Furthermore, this group has delivered BACE siRNA in brain neuronal cells and the mouse brain by blood-brain barrier (BBB)-permeable rabies virus glycoprotein (RVG)-conjugated PMT, thus resulting in successful brain gene therapy [123]. The osmotically-active RVG-PMT has been found to enhance the receptor-mediated transcytosis by stimulation of the CME.

Therefore, the osmotic-activity-induced cellular uptake regulation is one way to provide highly promising stimuli-regulated smart polymeric systems.

### 3.6. Other Strategies Involved in Internal Stimuli-Responsive Systems

#### 3.6.1. Thermo-Responsive Systems

Temperature-responsive systems as physical stimuli have attracted much attention for developing stimuli-responsive gene carriers. These systems show a sharp change in physical properties, such as gelation after a small or moderate change between room temperature to body temperature (37 °C). Various in vitro applications have used this change in surface properties to mediate swelling and collapse of the polymer system to increase transfection efficiency. The difference in noticeable temperature variations in some disease/pathological conditions, such as the temperature increase (1–2 °C) in the tumor microenvironment compared with normal healthy tissues, have also been used in a thermo-responsive system [124].

Temperature-sensitive polymers and co-polymers have a specific critical solution temperature, and with small temperature changes around this temperature, the hydrophilic and hydrophobic interactions within the polymeric chain and the aqueous medium change. This change results in the disruption of intermolecular interactions, such as electrostatic and hydrophobic interactions, and thus, a volume phase transition, such as expansion or collapse, occurs [125]. Temperature-sensitive polymers have a specific critical solution temperature range, which is set by the upper critical solution temperature (UPST) or lower critical solution temperature (LCST). Those polymers that become insoluble after heating have an LCST, and those that become soluble after heating have a UCST. LCST and UCST systems restricted to the aqueous environment are important in the development of responsive gene carriers [126,127]. The sensitivity depends primarily on the hydrogen bonding interactions between the polymer chain and water. As the temperature increases, the efficiency of hydrogen bonding is inadequate for the polymer’s solubility in water, and as the temperature increases beyond the lower critical solution temperature (LCST), polymer chains change to insoluble globules (phase separation) [127,128].

Some of the temperature-responsive polymers include poly(*N*-alkyl substituted acryl amides), poly(*N*-vinyl alkylamides), co-polymers, such as PEG/poly(lactide-*co*-glycolide) (PEG/PLGA), triblock copolymers, such as polyoxyethylene-polyoxypropylene-polyoxyethylene (PEO-PPO-PEO), and poly(ethylene glycol)-poly(lactic acid)-poly(ethylene glycol) (PEG-PLA-PEG) [129,130,131]. Among them, poly(*N*-isopropylacrylamide) (poly(NIPAAM)), a poly(*N*-alkyl substituted acryl amide), has been extensively studied. It is soluble in water below 32 °C (LCST) and changes to an opaque gel form above this temperature. This polymer can be used for controlled delivery, because a therapeutic drug-incorporated polymer turns into a gel. The sol/gel transition temperature of the polymer can be fine-tuned by the copolymerization of hydrophobic moieties or by controlling the molecular weight of the polymer. The conjugation of hydrophilic groups such as acrylic acid with the polymer also increases LCST to 37 °C [11,132].

Hennik et al. have copolymerized *N*,*N*-dimethylaminoethyl methacrylate (DMAEMA) with NIPAAM to develop a gene carrier system by making stable polymer/plasmid complexes. The complexes prepared at 25 °C are relatively stable at 37 °C and show high transfection efficiency in a human ovarian cancer cell line (OVCAR-3). The authors have also examined the effects of composition and the molecular weight of the polymers on transfection efficiency [133]. Kurisawa et al. have developed a water-soluble thermo-responsive copolymer, poly(*N*-isopropylacrylamide (IPAAm)-*co*-2-(dimethylamino) ethyl methacrylate (DMAEMA)-*co*-butyl methacrylate (BMA)), as a gene carrier. The polyplexes have been found to exhibit a complete temperature dependence on gene transfer efficiency in COS-1 cells, as determined by using a β-galactosidase reporter gene. The LCST of the copolymer is maintained at an applicable level for the cells by the BMA units in the polymers [134].

Most of the thermosensitive polymers consist of a cationic segment, such as polyarginine, PEI, PLL, chitosan, and a thermosensitive polymer, such as PNIPAAM or its derivatives. Thermosensitive submicron particles have been developed by copolymerization of NIPAAM with a cationic co-monomer, such as *N*-3-dimethylaminopropylmethacrylamide (DMAPM), which has a high ionization capability for plasmid DNA delivery [135]. Another thermosensitive polymer is PEO-PPO-PEO (trade name Pluronic), which is in fluid form at room temperature and converts to a gel state at body temperature (37 °C) [136]. Choi et al. have developed temperature-sensitive PEO-PPO-PEO/PEI nanocapsules for triggered endosomal compartment disruption at body temperature [137]. A tumor-targeted gene delivery system developed by cross linking Pluronic 123 with PEI and modified with a targeting peptide has been developed for enhanced transfection efficiency [138]. Many other nanocarriers, including hydrogel systems based on thermosensitive polymers, have been developed for effective gene delivery application by cold shocks or hyperthermia-mediated photo-thermal therapy, although these systems are beyond the scope of this review.

#### 3.6.2. Hypoxia- and Inflammation-Induced Gene Delivery Systems

Apart from the stimuli-responsive systems explained above, some other stimuli-mediated carrier systems based on stimuli such as hypoxia and inflammation have been developed. A nanocarrier PAPD made from poly(ethylene glycol)  (MW: 2000), azobenzene, polyethyleneimine (PEI) (1.8 kDa) and 1,2-dioleyl-*sn*-glycero-3-phosphoethanolamine (DOPE) units, has been used for hypoxia-induced siRNA uptake and silencing. Azobenzene has been used as a hypoxia-reactive bioreductive linker for hypoxia-mediated gene delivery. After the PEG removal/cleavage, siRNA is released from the PEGylated nanopreparations. Hypoxia-activated silencing of green fluorescent protein (GFP) in in vitro and in vivo experiments have shown effective silencing, and thus, this nanoformulation can be further used for siRNA delivery [139]. Another strategy is the inflammatory response-mediated system. Inflammatory responses are mediated by immune cells, such as T and B lymphocytes, followed by polymorpho nuclear (PMN) leukocytes and macrophages. This process involves the production of various kinds of molecules and enzymes. Various reactive oxygen species, such as oxygen-free radicals, which are released during inflammatory reactions, can be utilized for developing inflammation-responsive carrier systems [140].

An increased amount of glucose leads to diabetes mellitus and associated kidney and heart dysfunction. A glucose-responsive carrier system has been used for insulin delivery in a controlled manner. A similar approach may also be applied for an efficient glucose-responsive gene delivery in the future [141].

#### 3.6.3. Multi-Stimuli-Responsive Gene Carriers

For enhanced drug/gene delivery applications, many multi-stimuli-responsive systems incorporating more than one stimulus-responsive component with more specificity and versatility have been developed. Some of the multi-stimuli-responsive systems developed for gene delivery are briefly explained in this section. Recently, a highly efficient polymeric nanocomplex system with sequential activation by quadruple stimuli has been introduced. The system includes a photosensitizer (pheophorbide A (PhA))-loaded thiol-degradable polycation (high molecular weight (HMW)), bioreducible poly (ethylenimine (RPC)) and cytomegalovirus (CMV) promoter-equipped pDNA to form a PhA@RPC/pDNA complex. In the prepared PhA@RPC/pDNA complexes, the PhA@RPC gene carrier responds to pH, thiol and light, and the genes with CMV promoters are stimulated by ROS-activated NF-κB. The uptake and further activation of the system is estimated to be 2 h for cell uptake, 4 h for endosomal escape and 12 h for NF-κB-triggered CMV activation. This system shows negligible toxicity and enhanced transfection efficiency in MDA-MB-231 cells [142]. A redox-responsive and acidity-accelerative decomposition-mediated supramolecular gene vector (SS-OPP) based on three building blocks, including a disulfide-expanded PEG chain (SSPEG), an α-cyclodextrin (α-CD) moiety post-modified with oligoethylenimine (OEI 600) and a fluorescent conjugate (PBA-ARS) linked with an acid-cleavable phenylboronate bond between phenylboronic acid (PBA) and bulky alizarin red (ARS) for end-capping the polyrotaxane, has been developed. This polymer allows for very rapid gene accumulation in the nuclei of tumor cells. The SS-OPP decomposes in the high GSH stimuli in cancer cells, and this decomposition is followed by removal of the ARS group, charge repulsion and phenyl boronate cleavage. Compared with PEI25, the gold standard for non-viral vectors, this system shows enhanced transfection efficiency in in vitro and in vivo conditions [143]. By utilizing a cell-penetrating peptide (designated as dtACPP), a dual-triggered microenvironment-responsive delivery system has been developed and demonstrated to respond to lowered pH (tumor extracellular pH) and MMP-2. In the tumor environment, the dtACPP is activated, and the cell-penetrating peptides are exposed, thus driving the internalization of the nanoparticles into the intratumoral cells and co-delivering the plasmid and DOX. A short interfering RNA targeting VEGF (shVEGF) and DOX has been found to effectively shut down the blood vessels and to promote tumor cell apoptosis [144]. A poly(cyclodextrin) (PCD)-based, reduction and light dual stimuli-responsive supramolecular host-guest self-assembly system with disulfide containing azobenzene-terminated branched poly(2-(dimethylamino)ethyl methacrylate) (Az-ss-BPDMs) was developed for gene delivery. The Az-ss-BPDMs showed good DNA binding and condensation property. The supramolecular polycations PCD/Az-ss-BPDM and their polyplexes PCD/Az-ss-BPDMs/DNA showed good reduction and photo-sensitiveness with high gene transfection efficiency and low toxicity compared to those in the absence of PCD. The branching degree and the introduction of disulfide bond also showed a positive correlation with the gene transfection efficiency [145]. Table 4 summarizes some of the ROS, enzyme or multi-stimuli-responsive polymer systems used for gene delivery applications.

## 4. Conclusions and Future Perspectives

Even though conventional therapeutic systems are advantageous, the changing metabolism of the body makes this approach less sensitive. A self-regulated gene delivery mechanism adjusted to different biological rhythms is beneficial for the controlled release of therapeutics in a spatially- and temporally-controlled manner. Among internal and external stimuli, the internal stimuli represent a unique signature for many pathological conditions and, thus, are an attractive trigger for localized gene delivery. Although stimuli-responsive delivery systems have emerged only recently, they have already demonstrated great potential in biomedical applications. The response of a stimuli-responsive system can occur through the use of a stimuli-responsive polymer or through combining a stimuli-responsive compound with a normal polymer backbone, and the response of the polymer can be either a structural alteration or a change in its properties. Various internal stimuli-responsive polymers and delivery systems have been developed for therapeutic purposes and have been discussed in this review.

Even though numerous stimuli-responsive gene carriers have been developed for biomedical applications, several challenges remain to be addressed to allow for realistic applications; such challenges include the safety of the material or their modifying moieties, toxicity, variations in different disease/pathological conditions with different levels of the stimuli and variations in in vitro and in vivo efficiency. In the future, these clinical devices may allow for the release of a gene into specific tissues in an efficient and controlled manner, an outcome that would scarcely be achievable though other means. Future studies should also focus on identifying single systems by integrating many applications with synergistic outcomes.

## Figures and Tables

**Table 1:** pH-sensitive delivery systems for therapeutic gene delivery.

Polymer used	pH-responsive moiety	Drug used	Ref.
PEG-lipid:DOPE liposomes	Phenyl-substituted vinyl ether (PIVE)	Plasmid DNA	[54]
Lipid-coated PBAE nanoparticles	Poly(β-amino ester) (PBAE)	mRNA	[55]
Polymerized diacetylenic amphiphile (PDA) micelles	Imidazole group of histidine	siRNA	[56]
Poly(2(dimethylamino) ethyl methacrylate)-block poly(2(diisopropylamino)ethyl methacrylate) (PDMA-*b*-PDPA) micelles	PDPA protonation	siRNA and Amphotericin B	[57]
PEG-pImHeMA-pGMA copolymer (polymersome)	Polyimidazole-hexyl methacrylate (poly-ImHeMA))	siRNA	[58]
Monomethoxy poly(ethylene glycol)-*b*-poly(amino acid methacryloyloxyethyl ester) (mPEGn-*b*-P(H2N-AA-EMA))	Poly((meth)acrylamide)	Plasmid DNA	[59]
Poly(dimethylaminoethyl methacrylate-block-butyl methacrylate) copolymers (pDbB) (micelle)	Poly(styrene-alt-maleic anhydride) (pSMA)	siRNA	[60]
PEI and hexanoate-PEI	Arginine rich peptides	pDNA, siRNA	[61]
Poly(*N*-((2-(2-(dimethylamino)ethoxy)-1,3-dioxolan-4-yl)methyl)methacrylamide (PMAOE)	Acid-cleavable side chain	pDNA	[62]

**Table 2:** Examples of redox-stimuli responsive gene delivery systems.

Polymer used	Type of system	Gene used	Ref.
Disulfide-containing cross-linked polyethylenimines (PEI-SS-CLs)	Polyplexes	pDNA	[84]
Hyaluronic acid coated reducible hyperbranched poly-(amidoamine) (RHB) (HA/RHB/pDNA)	Nanoassembly	pDNA	[85]
Hyperbranched PAAs with tertiary amino cores and amine, poly(ethylene glycol) (PEG) and hydroxyl terminal groups	Polyplexes	pDNA	[86]
mPEG-*b*-PLL-*g*-(ss-lPEI) (PLI)	Polyplexes	XIAP siRNA	[87]
Bock co-polymer from PLGA-*s*-*s*-PEGMA	Core-shell nanoparticle	pDNA	[88]
Polyaspartamide-based disulfide-containing brushed polyethylenimine (P(Asp-Az)X-SS-PEIs)	Polyplexes	pDNA	[89]
Reducible copolypeptides (rCPP) with HRP(histidine-rich peptide (HRP)) and NLS sequences	Polyplexes	p DNA	[90]
SSPEI	Polyplexes	siRNA	[91]
Arginine-conjugated poly(cystamine-*bi*s-acrylamide-diaminohexane) (poly(CBA-DAH-R))	Polyplexes	siRNA	[92]

**Table 3:** Osmolarity (mOsm) of polyplexes Reproduced with permission from [122]. Copyright proceedings from Elsevier.

RPMI + 10% FBS		276
Mannitol	1%	348
	3%	441
	5%	531
PMT/DNA (N/P 20)	1% mannitol (wt %)	297
	3% mannitol (wt %)	395
	5% mannitol (wt %)	421

**Table 4:** ROS, enzyme and multi-stimuli responsive polymer preparations used for gene delivery.

Polymer used	Type of trigger	Type of linkage/sensitive moieties	Gene used	Ref.
Poly-(1,4-phenyleneacetone dimethylene thioketal)	ROS	Thioketal	siRNA	[146]
mPEG113-*b*-CP5K-*b*-PDMAEMA42-*b*-P(DMAEMA22-*co*-BMA40-*co*-PAA24) (PPDDBP)	ROS and pH	CP5K peptide linker, PAA	pDNA	[147]
PEG2000-peptidyl lipids	Enzyme	Elastase or MMP-2-mediated digestion	pDNA	[148]
PEG-pp-PEI-PE	Enzyme	MMP-2-mediated digestion	siRNA	[149]
PEG-PAsp(AED)-PDPA	pH and redox potential	Di-sulfide linkage	siRNA	[150]
